# Analysis of culling reasons during the breeding cycle and lifetime performance: The strategy to remove crossbred Landrace and Large White sows under tropical climate

**DOI:** 10.14202/vetworld.2021.3170-3174

**Published:** 2021-12-26

**Authors:** Ratchadaporn Boripun, Watcharapong Mitsuwan, Pawinee Kulnanan, Thotsapol Thomrongsuwannakij, Warangkana Kitpipit

**Affiliations:** 1 Akkhraratchakumari Veterinary College, Walailak University, Nakhon Si Thammarat, 80160, Thailand; 2 One Health Research Center, Walailak University, Nakhon Si Thammarat, 80160, Thailand; 3 Research Center of Excellence in Innovation of Essential Oil, Walailak University, Nakhon Si Thammarat, 80160, Thailand; 4 Food Technology and Innovation Center of Excellence, Walailak University, Nakhon Si Thammarat, 80160, Thailand

**Keywords:** culling, parity, season, sow, tropical climate

## Abstract

**Background and Aim:**

Sow culling is an important practice in commercial swine production because it is directly associated with the economic efficiency of the breeding herd. This study was conducted to analyze the reasons for sow culling and quantify the factors affecting culling in crossbred Landrace and Large White sows under tropical climate.

**Materials and Methods:**

A total of 4887 culled sows from one parent stock farm located in Ratchaburi province, Western Thailand, were examined in this study. Culling reasons were grouped into the following eight categories according to farm management: (1) Reproductive disorders, (2) old age, (3) low performance, (4) diseases, (5) lameness, (6) udder problems, (7) body condition, and (8) other illnesses. Logistic regression analysis was used to explore the relationship between culling sows and environmental factors. Effects of parity and season of culling were considered as fixed effects in a statistical model.

**Results:**

Descriptive statistics indicated the following factors accounting for sow removals: Old age (34.93%, n=1707), reproductive disorders (29.32%, n=1433), low performance (12.62%, n=617), lameness (12.56%, n=614), diseases (4.8%, n=235), body condition (4.68%, n=229), udder problems (0.79%, n=39), and other illnesses (0.26%, n=13). Parity and season of culling were also found to have a significant effect on sow culling (p<0.05). The majority of culling sows in this population were of old age and high parity.

**Conclusion:**

This study indicated that the purposeful culling of sows on this farm was within the targeted range. However, the incidence of reproductive disorders was too high and required further investigations.

## Introduction

In commercial swine production, sow culling is an important practice because it is directly related to the economic efficiency of the breeding herd [1]. A good culling policy with a sound understanding of the culling time is an integral component of herd management. It maintains a steady flow, replacing the less productive sows on a regular basis, without disrupting the overall performance of the breeding herd. The number of piglets and the lifetime performance of the breeding sow are the primary aspects of swine economic value. Therefore, the high performance and longevity of breeding gilts and sows are the most important parameters for profitability in swine production [2]. Although sow performance is important to increase the number of piglets, the wastage of swine feed is also considered in the farm industry. Therefore, appropriate culling of gilts and sows is one of the possible strategies to address this issue [1]. The annual culling rates in the swine industry in several countries, including Japan, Spain, Sweden, and USA, were reported to be 35.7-49.5% [3,4]. Several reasons, including common diseases, reproductive system diseases, and low milk production, have been used as criteria for the culling of gilts and sows [1].

Swine breeding techniques often consider production traits that stimulate animal growth. Moreover, the techniques are powerful to increase lean meat and decrease production costs [5]. The crossbred Landrace and Large White are some of the most popular gilts and sows available in several countries, including Thailand [6,7]. It has been reported that these crossbred sows produce a number of piglets with a high percentage ofsurvival [8]. They also have the good maternal ability.

Ratchaburi province is located in a tropical climate zone, and its latitude and longitude coordinates are 13°32’12.16”N, 99°49’1.63”E. It is one of the leading areas of swine production in Thailand. The climate in Southeast Asia, including Thailand, is hot, rainy, and wet environment [9]. Several factors such as parity and season affect the culling of sows. Seasonal variations could influence fertility, feed intake, and heat stress, and different parity may affect reproductive ability. Gilts and sows have different hormone systems, milking ability, maturity, and pregnancy experience. Therefore, the fertilization rates of sows may decrease. Several parameters of culling, including diseases, milk production, reproductive system, and season of the tropical climate, are used as criteria for the culling of gilts and sows. All this information was statistically analyzed to evaluate the culling reason.

Therefore, the objectives of this study were (1) to analyze the reasons for sow culling and (2) to quantify the factors influencing the culling pattern in crossbred sows and gilts (Landrace×Large White) under tropical climate.

## Materials and Methods

### Ethical approval

Animal Care and Use Committee approval was not obtained for this study because the information was retrieved from the database of a single large integrated swine production company and animals were not directly used for the study.

### Study period and location

The study data were collected from 2012 to 2015. The farm was located in Ratchaburi province, Western part of Thailand.

### Herds and housing systems

The study was focused on a single large integrated swine production company’s large-scale indoor swine breeding unit, which is located in Ratchaburi province, Western Thailand. The data included in the present study were derived from crossbred gilts and sows (Landrace×Large White) of the commercial farm. They were mated with Duroc sires to produce fattening pigs. Finally, the genotype of the fattening pigs contained 25% Landrace, 25% Large White, and 50% Duroc. Gilts and sows were reared under an evaporative cooling system. On this farm, a gestating house was equipped with an automatic feeding system. The amounts of feed for the gilts and sows were 2.0-3.0 and 3.0-3.5 kg/day, respectively. The gilts and sows were housed individually in stalls with slatted flooring.

### Data collection and classification of culling reasons

Retrospective data were captured in theFinLIVE database using the information of culled sows. The gilts and sows included in the present study were crossbreds between Landrace and Large White. Data on a total of 4887 sows were collected to investigate culling analysis. Data collection was performed by recording the initial culling data of gilts and sows from the farmers of the herds since 2012 and 2015. Culling reasons were identified and classified into eight major categories (Table-1).

**Table 1:** The culling reason are divided into categories.

Culling reason	Components
Reproductive disorder	Repeated returns, anestrous, abortion, did not conceive
Old age	old age
Low performance	Poor litter size, poor lactation, and rearing ability, poor maternal behavior, farrowing difficulties
Disease	Respiratory disease, gastrointestinal disease
Lameness	Dog sitter, foot pain, foot swelling, hoof crack, lameness, paralysis
Udder problems	Low or no milk production, mastitis and/or udder abscess
Body condition	poor body condition, unthrifty
Other illnesses	Stress, fever, neurological disorders, prolapse, sudden death, unknown

Moreover, the effects of season on the culling of gilts and sows were evaluated in this study. According to the weather in Thailand, the periods of the season were defined as summer (February-May), rainy (June-September), and winter (October-January) [10].

### Statistical analysis

The collected information was first examined using descriptive analysis to describe the reasons for the culling of individual sows. Next, logistic regression analysis was used to investigate the effects of each reason on the culling. All analytical procedures were conducted using R statistical software (R Core Team, 2020) [11]. A significant difference was presented if p<0.05.

## Results

### Sow culling reasons

A total of 4887 sows reared under the evaporative cooling system were used to identify the culling reasons using eight primary types, including reproductive disorders, old age, low performance, diseases, lameness, udder problems, body condition, and other illnesses. The major reason for sow culling was old age (1707 sows, 34.93%), followed by reproductive disorders (1433 sows, 29.3%) and low performance (617 sows, 12.62%) (Table-2). However, the lowest frequency of culling reasons was observed for other illnesses (13 sows, 0.26%), followed by udder problems (39 sows, 0.79%) and body condition (229 sows, 4.68).

**Table 2:** The number and frequency (%) of sow culling by removal reasons.

Culling reason	Number of sows (%)
Old age	1,707 (34.93)
Reproductive disorders	1,433 (29.32)
Low performances	617 (12.62)
Lameness	614 (12.56)
Diseases	235 (4.80)
Body condition	229 (4.68)
Udder problems	39 (0.79)
Other illnesses	13 (0.26)
Total	4,887 (100)

### Sow culling by parity

Eight parity distribution of sow culling was evaluated from the total of 4887 culled gilts and sows in this study. As shown in Table-3, the 7^th^ parity accounted for the highest proportion of culled sows (19.35%), followed by the 1^st^ (15.83%) and 6^th^ (12.23%) parities. In contrast, the lowest frequent parity of culled sows was parity numbers 8 (3.76%), 4 (7.73%), and 3 (8.24%).

**Table 3:** The number and frequency (%) of sow culling by parity.

Culling reason	Parity (%)									

	0	1	2	3	4	5	6	7	8	Total
Old age	0 (0)	0 (0)	0 (0)	10 (0.20)	17 (0.34)	145 (2.96)	457 (9.35)	901 (18.43)	177 (3.62)	1,707 (34.93)
Reproductive disorders	476 (9.74)	324 (6.63)	190 (3.88)	140 (2.86)	132 (2.70)	120 (2.45)	42 (0.86)	9 (0.18)	0 (0)	1,433 (29.32)
Low performances	25 (0.51)	147 (3.00)	227 (5.67)	76 (1.55)	58 (1.18)	50 (1.02)	21 (0.43)	11 (0.23)	2 (0.04)	617 (12.62)
Lameness	37 (0.76)	176 (3.60)	89 (1.82)	85 (1.74)	94 (1.92)	84 (1.72)	35 (0.72)	13 (0.27)	1 (0.02)	614 (12.56)
Diseases	4 (0.08)	58 (1.19)	30 (0.61)	32 (0.65)	47 (0.96)	46 (0.94)	10 (0.20)	8 (0.16)	0 (0)	235 (4.80)
Body condition	12 (0.25)	58 (1.19)	33 (0.67)	55 (1.13)	30 (0.61)	16 (0.33)	21 (0.43)	4 (0.08)	0 (0)	229 (4.68)
Udder problems	0 (0)	6 (0.12)	12 (0.24)	3 (0.06)	0 (0)	4 (0.08)	10 (0.20)	0 (0)	4 (0.08)	39 (0.79)
Other illnesses	0 (0)	5 (0.10)	2 (0.04)	2 (0.04)	0 (0)	2 (0.04)	2 (0.04)	0 (0)	0 (0)	13 (0.26)
Total	554 (11.33)	774 (15.83)	583 (11.02)	403 (8.24)	378 (7.73)	467 (9.55)	598 (12.23)	946 (19.35)	184 (3.76)	4,887 (100)

Table-3 also shows the proportion of culling according to the classification of culling reasons within each parity. Old age was found to be the major culling reason at parity 7, followed by parity numbers 6 and 8. Furthermore, reproductive disorders were found to be an important culling reason in the population. Most sows with reproductive disorders were removed since parity numbers 0, 1, and 2, respectively. A total of 476 sows were culled at parity 0 due to reproductive disorder conditions. The lowest frequency of culling was observed for other illnesses.

### Sow culling by season

The variation in the seasonal periods of Thailand affects on swine productivity and it is well known that seasonal periods affect the fertilization of swine. Therefore, the effects of seasons on the culling of sows were analyzed in this study. Results showed that most sows were culled in the rainy season, followed by winter and summer seasons (Table-4), with the proportions of culled sows being 36%, 34%, and 30%, respectively.

**Table 4:** The number (percentage) of sow culling by season.

Culling reason	Summer (%)	Rainy (%)	Winter (%)	Total (%)
Old age	436 (8.92)	601 (12.29)	670 (13.70)	1,707 (34.93)
Reproductive disorders	427 (8.74)	604 (12.36)	402 (8.22)	1,433 (29.32)
Low performances	187 (3.83)	178 (3.64)	252 (5.15)	617 (12.62)
Lameness	198 (4.05)	249 (5.09)	167 (3.41)	614 (12.56)
Diseases	85 (1.74)	84 (1.72)	66 (1.35)	235 (4.80)
Body condition	72 (1.47)	118 (2.42)	39 (0.79)	229 (4.68)
Udder problems	19 (0.39)	18 (0.37)	2 (0.04)	39 (0.79)
Other illnesses	2 (0.04)	8 (0.16)	3 (0.06)	13 (0.26)
Total	1,426 (29.18)	1,860 (38.06)	1,601 (32.76)	4,887 (100)

In the rainy season, reproductive disorders and old age were found to be the major reasons for culling, accounting for 604 (12.35%) and 601 (12.30%) sows, respectively, followed by lameness (249 sows, 5.10%). In the summer and winter seasons, the highest proportion of sow culling was due to reproductive disorders (565 sows, 11.56%). In addition, old age was the second important culling reason in both summer and winter seasons, with 436 (8.92%) and 670 (13.71%) culled sows, respectively.

### Factors influencing sow culling

All data were analyzed to examine the effect of season and parity on sow culling. Data concerning the season included summer, rainy, and winter. All parities of sow culling were evaluated to determine the association with all culling reasons. Results showed that the seasons of culling and parity were significantly associated with all culling reasons (p<0.05).

## Discussion

The present study is one of the few investigations on the culling pattern of gilts and sows in Ratchaburi Province, a region with a tropical climate and one of the key areas of swine industry development in Thailand. The crossbreeds of Landrace and Large White were selected as the representative sample as they are one of the most popular gilts and sows available in several countries, including Thailand [6,7]. Moreover, these crossbred sows produced a number of piglets with a high percentage of livability [8]. It is well known that sow culling rates are directly related to the economic efficiency of the breeding herd. Therefore, this investigation has revealed the reasons for the culling of crossbred Landrace and Large White sows under tropical climate. The highest proportion of sow culling was observed for parity number 7, followed by 1 and 6. The optimum average herd life to maximize herd productivity has been estimated between 5 and 10 parities [12]. In contrast, the lowest proportion of sow culling was observed for parity numbers 8, 4, and 3. According to Koketsu *et al*. [13], sow-level predictor analysis showed that the lower age at first mating was associated with higher lifetime performance [2]. Other examples were that lack of repeat mating in parity 0 and shorter weaning-to-first-mating interval in parity 1 was associated with higher fertility, whereas more piglets born in parity 1 were associated with higher prolificacy. Dhliwayo [14] classified sow culling into two groups of planned and unplanned sow culling. Planned sow removal was a common practice in commercial pig production, which was attributed to old age and low performance. Unplanned culling was the other type of sow removal attributed to reproductive disorders, diseases, lameness, udder problems, body condition score, and other illnesses. The major reason for sow culling was old age, followed by reproductive disorders and low performance. Similarly, old age was found to be the major reason for sow culling associated with on-farm production records in USA [15]. It has been reported that the length of productive life and lifetime production of sows is a crucial consideration in the commercial swine production farm [16]. The primary causes of reproductive disorders were poor farm management, high outdoor temperature, malnutrition, poor inseminations, and new experience of workers. Low performance was induced from increased lactation length, prolonged weaning-to-first-mating interval, increased number of stillborn piglets, foster-in or nurse sow practices and low or high age at first mating, and low birth weight or low preweaning growth rate. Sows that began to farrow at younger ages had a significantly longer length of productive life, a higher number of lifetime piglets born alive, a higher number of lifetime piglets weaned, heavier lifetime piglet birth weight, and heavier lifetime piglet weaning weight than sows that farrowed at older ages [16]. It has also been documented that higher age at first mating decreases longevity and lifetime reproductive efficiency of sows in breeding herds [16].

Similarly, the results of this study showed that the highest parity of culled sows was 7, which was the major reason for culled sows coming from old age. It has been reported that piglet survival rates declined from parity 7. In addition, it has been reported that the litter weight and survival rate of piglets born from parity 7 were decreased [17]. Moreover, a previous study suggested that the possibility of late pregnancy loss increased with increasing parity, increasing weaning-to-service interval, decreasing lactation length, and decreasing weaned litter size [15].

Regarding the effects of season on the culling of sows, the highest proportion of sow culling was observed in the rainy season, followed by winter and summer seasons in the present study. Stankiewicz *et al*. [18] also reported the influence of seasons on the ovarian cycle and fertilization of swine. However, the proportion of sow culling may also be affected due to other seasons; for example, the high culling rate in the rainy season might be causing lower culling rates in winter and summer seasons. Reproductive disorders were the major reason for sow culling in the rainy season, followed by old age and diseases. This may be due to the farm management or winter and summer environments. The reasons for sow culling were significantly influenced by all parties and seasons. Therefore, the differentiation of parity and seasonal conditions affected sow culling and lifetime sow productivity in this commercial farm. Because the season condition influenced the diversity of environmental conditions such as farm management, nutrition, temperature, humidity, and healthcare, this aspect must be considered in farm management for improving sow lifetime productivity in this commercial farm.

## Conclusion

This study analyzed the reasons for the culling of crossbred gilts and sows (Landrace×Large White) under tropical climate, wherein the major reason for sow culling was identified as old age followed by reproductive disorders and low performance. The culling was highest in parity 7, followed by 1 and 6. However, the lowest culling proportions were noted in parity numbers 8, 4, and 3, respectively. Most sows were culled in the rainy season, followed by winter and summer. In the rainy season, reproductive disorders (12.35%) and old age (12.30%) comprised the major reasons for culling, followed by lameness. These data could be used as a model to investigate sow culling in pig farms in tropical areas. Subsequently, our results could also be used for the development of farm management, leading to improvement in sow lifetime productivity in such areas.

## Authors’ Contributions

RB, WM, and WK: Conceived and designed the experiments. RB, WM, and WK: Collected the data, analyzed and interpreted the data. RB, WM, PK, TT, and WK: Wrote and corrected the manuscript. All authors read and approved the final manuscript.
